# The role of VEGF and TGF-β blood levels for fibroid shrinkage, menorrhagia score, and quality of life improvement after uterine artery embolization for uterine fibroids: a study protocol

**DOI:** 10.3389/fmed.2024.1382822

**Published:** 2024-08-06

**Authors:** Gulzhanat Aimagambetova, Gauri Bapayeva, Talshyn Ukybassova, Viktor Zemlyanskiy, Arnur Gusmanov, Milan Terzic

**Affiliations:** ^1^Department of Surgery, School of Medicine, Nazarbayev University, Astana, Kazakhstan; ^2^Clinical Academic Department of Women’s Health, CF “University Medical Center”, Astana, Kazakhstan; ^3^Clinical Academic Department of Radiology and Nuclear Medicine, CF “University Medical Center”, Astana, Kazakhstan; ^4^Department of Biomedical Sciences, School of Medicine, Nazarbayev University, Astana, Kazakhstan

**Keywords:** menorrhagia, quality of life, uterine artery embolization (UAE), uterine fibroids, fibroid shrinkage, VEGF, TGF-**β**

## Abstract

Uterine leiomyoma is a common condition affecting women that occurs in more than 70% of females. Women with leiomyomas overall have lower quality of life and deficiency in many specific spheres of life including work-related productivity, sexuality, relationships, social–emotional health, and physical well-being that might be present even in pre-and extended throughout early postmenopausal life. Choices for symptomatic fibroid management include medical, interventional radiology procedures, surgical, and expectant management. The uterine artery embolization (UAE) procedure has gained justified popularity for myoma management. Growth factors, vascular endothelial growth factor (VEGF) and tumor growth factor β (TGF-β), hold an important role in leiomyoma progression. However, blood levels of VEGF and TGF-β in patients before and after UAE are not fully investigated and the possible relationship with myoma shrinkage has not been evaluated. Therefore, this study aims to assess menorrhagia score and quality of life improvement after UAE for uterine fibroids and compare blood levels of VEGF and TGF-β in patients with uterine leiomyoma before and after UAE. This cross-sectional study will be performed at the University Medical Center, Astana, Kazakhstan. Women undergoing the UAE procedure for uterine leiomyoma will be involved in the study following the precisely defined inclusion/exclusion criteria. Uterine leiomyoma nodules’ structural changes after UAE will be assessed along with the blood levels of growth factors (VEGF and TGF-β), menorrhagia score, and quality of life. An important outcome of this project will be an investigation of the blood levels of growth factors (VEGF and TGF-β) before and after the procedure and their association with leiomyoma shrinkage in correlation with the menorrhagia score and quality of life alterations among patients undergoing UAE.

## 1 Introduction

Uterine leiomyoma (myoma or fibroid), is the most continual benign mass of the uterus, which occurs in more than 70% of women (1). Hysterectomy is still the most frequently offered and performed intervention to treat uterine leiomyoma (2). However, surgical management may lead to complications and negatively affects the patients’ quality of life (3, 4). Over the past three decades, a minimally invasive approach has been acquiring popularity. Uterine fibroids embolization became a typical option for many patients who are looking for an organ-sparing procedure (5–7). Since the implementation, strong evidence has been constructed presenting safety and efficacy of this approach and low rates of complication and side effects (5, 8).

Uterine artery embolization (UAE) was initially defined as a therapeutic option for symptomatic uterine fibroids in 1995 and became very popular in the last two decades (9). The advantages of UAE over surgery are a short hospital admission period, a quick return to normal life activities (usually 1–2 weeks), a low rate of major complications, the opportunity for uterine/fertility-sparing procedure, and the overall better quality of life in most of the cases (5, 10). The goal of UAE is to decrease blood flow to fibroid nodules that will cause ischemia/infarction and shrinkage of the uterine leiomyoma nodes (fibroids). It is an efficient and effective method as it reduces uterine bleeding in up to 85% of the cases and decreases pain in up to 80% of the patients treated with this method.

The latest Royal College of Obstetrics and Gynecology (RCOG) and American College of Obstetricians and Gynecologists (ACOG) guidelines recognized UAE as effective as surgery for control of symptoms and recommended the procedure for patients with symptomatic uterine leiomyoma (11, 12). The Cardiovascular and Interventional Radiological Society of Europe (CIRSE) standards of practice also recognized UAE as a “true alternative to hysterectomy in women who wanted to preserve their uterus” (13). However, previous studies confirmed that within the following 5 years after UAE due to the uterine leiomyoma relapse, 10% of patients may require a hysterectomy, 3% - myomectomy, and 2% will require repeating the UAE procedure (14–17).

Uterine leiomyoma development is associated with the involvement of a variety of growth factors. Those are proteins or polypeptides, which are produced by several cell types (18). They have a variety of biological functions/effects and generally act via an autocrine and/or paracrine pathway. Growth factors effects on target cells by targeting specific receptors on a cell surface. The subsequent signaling transmits the effects of growth factors in the cell (14). The most important markers for uterine fibroid growth are vascular endothelial growth factor (VEGF) and transforming growth factor β (TGF-β) (14, 19, 20).

VEGF is a cytokine, which possesses multiple effects: increases microvascular permeability, and stimulates endothelial cells’ growth and angiogenesis. VEGF is synthesized and secreted by various tumor cells and tumors in humans. It is an essential growth factor that supports the vascular endothelial cells proliferation, promotes cell migration and proliferation, and regulate angiogenesis (14, 21). Thus, VEGF plays a significant role in the growth of uterine fibroids (22). Although uterine leiomyoma is a benign mass, angiogenesis is also critical for its existence and development. UAE procedure could induce uterine fibroids’ tissue ischemia and hypoxia via the blood supply obstruction. In the next step, hypoxia can prompt VEGF gene transcription, thus, elevating the expression level of VEGF and invigorating leiomyoma-related angiogenesis (14, 21, 23). According to the literature data, it is not clear and has to be elucidated further whether VEGF blood levels could be used as a prognostic factor for patients with uterine leiomyoma following UAE treatment (23).

TGF-β is known as a versatile cytokine with a dimeric polypeptide structure. TGF-β has three different isoforms and belongs to a large family of proteins entitled TGF-β superfamily (24). TGF-β regulates multiple inflammation mediators, cell division, and extracellular matrix production (24). Moreover, it plays a major part in cellular migration within a tumor, stimulates tumor growth, and enhances metabolism. Therefore, the synthesis and release of TGF-β in a fibroid nodule are increased, and results in excessive extracellular matrix production and deposition. According to available data, TGF-β substantially contributes to the growth and development of uterine leiomyoma by at least two mechanisms: (1) acts as an intermediary for sex steroid hormones, and (2) involvement in the processes of cell proliferation and migration, progression of fibrosis, and angiogenesis. TGF-β has meaningfully higher expression in uterine fibroid tissue than in normal myometrium (17). Furthermore, it has been specified that elevated levels of serum TGF-β are complemented by an elevated likelihood of uterine leiomyoma incidence (25). Recent investigation confirmed that inhibitors of VEGF and TGF-β can be used as therapeutic agents for uterine leiomyoma (19).

Currently, there is no data about the levels of VEGF and TGF-β in the peripheral blood of patients undergoing UAE, and their relationship with the uterine fibroid shrinkage. Moreover, blood levels of these growth factors in patients before and after UAE are not systematically investigated and eventual relationship with myoma shrinkage has not been evaluated. Therefore, this project aims to (1) assess menorrhagia score and quality of life improvement after uterine artery embolization for uterine fibroids and (2) compare blood levels of VEGF, and TGF-β in patients with uterine fibroids before and after UAE and to investigate their association with myoma shrinkage.

## 2 Methods

### 2.1 Study design and study setting

This study will utilize a three-year prospective cross-sectional study, including an 18-month recruitment period, data collection, laboratory processing, and post-study analysis. The study population will be drawn from women between the ages of 18–45 years with a history of uterine leiomyoma seeking UAE treatment, strictly following the inclusion and exclusion criteria. The patients will be recruited for the study from the gynecology and outpatient departments of the Clinical Academic Department (CAD) of Women’s Health, University Medical Center (UMC), Astana, Kazakhstan.

### 2.2 Study subjects

#### 2.2.1 Sample size justification

UMC outpatient department has an average of 2,000 outpatient visits per month and the Interventional Radiology Department performs 20–25 UAE per month (overall more than 250 procedures per year). To assess whether changes in blood levels of growth factors after UAE are correlated with alterations in the uterine fibroid’s volume, the required sample size is 120 patients. Using this desired sample size and formula indicated below, we computed achieved statistical power by setting Cohen’s d at 0.5, and significance level at 0.05. Calculations resulted in a statistical power of 99.97% for a two-tailed paired t-test. Null hypothesis: no statistical significance will exist in a set of given observations in blood levels of growth factors (VEGF, TGF beta) in patients before and after uterine artery embolization.


$$n={\left(\frac{2*{\left(Z_{\alpha /2}+Z_{\beta }\right)}^{2}}{d^{2}}\right)}^{2}$$

$Z_{\alpha /2}$ – Z score that corresponds to significance level ( α) for a two-tailed test.

$Z_{\beta }$ – Z score that corresponds to the desired statistical power $\left(1-\beta \right)$ for the test.

d – denotes the desired effect size (Cohen’s d), for which a moderate standardized difference (0.5) was taken.

#### 2.2.2 Patients evaluation

All patients scheduled for UAE will be evaluated by a gynecologist, an interventional radiologist, and an anesthesiologist. A detailed general and menstrual history and a thorough clinical evaluation will be performed to exclude other causes of menorrhagia. Magnetic resonance imaging (MRI) is proven to be the best imaging modality for evaluation of patients before and after UAE. MRI allows an accurate pre-procedure delineation of the vascular anatomy. Ultrasound (US) is an available and fast imaging modality to evaluate uterine fibroids. Both transvaginal and abdominal US scans will be required for a complex evaluation of the patient before the procedure. Although US imaging is a less sensitive modality for complete fibroid designation and delineation of possible concurrent pathologies within the uterus and adnexal regions, it allows a rapid evaluation, is less expensive, and is more feasible for patients’ selection.

#### 2.2.3 Inclusion and exclusion criteria

This project will strictly follow the inclusion and exclusion criteria.

##### 2.2.3.1 Inclusion criteria

Patients of 18–45 years old; history of symptomatic uterine intramural fibroids, or multiple symptomatic fibroids confirmed by visualization methods (ultrasound, magnetic resonance imaging) with severe menstrual bleeding and/or mechanical complaints/symptoms (pain, abdominal pressure, urinary urgency/frequency, dyspareunia); patient agrees with the method of treatment of uterine myoma – UAE.

##### 2.2.3.2 Exclusion criteria

Patients not fulfilling the inclusion criteria; asymptomatic women with uterine fibroids; women with isolated adenomyosis, cervical leiomyoma, pedunculated subserous and/or submucous leiomyoma, intraligamentous leiomyoma; leiomyoma larger than 10 cm in diameter; endometrial polyps and/or hyperplasia or presence of any malignancy; pregnant or breastfeeding women; the presence of any disease or medical condition that could worsen the patient’s safety and compliance in the study [chronic hypertension, cardiac insufficiency, renal insufficiency, diabetes mellitus (Type I or Type II), untreated hyper/hypothyroidism, hyperprolactinemia, chronic kidney disease, non-treated congenital of acquired cardiac pathology, multiple sclerosis, active vasculitis, uncontrolled coagulopathies]; pelvic irradiation history; contrast material allergy; concomitant use of GnRH analogs.

### 2.3 Basic angiographic and embolization techniques

Transcatheter embolization will be performed in an operating room with angiographic equipment in the Department of Interventional Radiology, UMC. Moderate sedation will be administered intravenously by an anesthesiologist to prevent pain and anxiety. The procedure for uterine artery embolization will be as follows: under local infiltration of anesthetic (Sol. Novocain 0.5% −20.0 mL/Sol. Lidocaine 1.0% −20.0 mL) puncture and catheterization of the right/left common femoral/radial artery by Seldinger will be performed. A 4- or 5-F selective angiographic catheter will be utilized for evaluation of the internal iliac artery, and a 2- or 3-F microcatheter will be used for super-selective catheterization of the uterine artery. The uterine artery originates from the internal iliac artery (anterior division), and it is well seen on the contralateral oblique projection. Robert’s Uterine Catheter (RUC) will be used to hook both uterine arteries. For embolization, microspheres and/or microparticles with a diameter of 500–1,100 μm will be used.

### 2.4 Growth factors analysis

The blood samples from the cubital vein will be centrifuged (15,000 r/m) and subject to deep freezing at –80°C (25). Storage of samples will be carried out in conditions of deep freezing. Transportation of blood samples to the laboratory of the Republican Diagnostic Center (part of UMC) will be carried out in a special container with refrigerants. The test values (TGF-β and VEGF) will be determined via the enzyme-linked immunosorbent assay (ELISA) using standard sets Multiplex MAP Human Cytokine/Chemokine Magnetic Bead Panel, Merck according to the manufacturer’s protocol on a Bio-Plex 3D flow cytometer. The expected values are given in the instructions attached to the kits. For the cohort of recruited patients (120), 480 blood samples will be analyzed (4 samples for each patient, overall 960 results for 2 growth factors) (Table 1).

**Table 1:** Timeline of procedures and investigations in the project.

Item/factor/procedure	Before UAE	1 week after UAE	1 month after UAE	3 months after UAE	1 year after UAE
UFS-QOL questionnaire	**+**	**−**	**−**	**+**	**+**
Menorrhagia score questionnaire	**+**	**−**	**−**	**+**	**+**
VEGF	**+**	**+**	**+**	**+**	**−**
TGF-β	**+**	**+**	**+**	**+**	**−**
US scan	**+**	**+**	**+**	**+**	**−**
MRI	**+**	**−**	**−**	**−**	**+**

### 2.5 Data collection procedures’ sequence

Before embolization of the uterine arteries, patients will be asked to fill out the questionnaire on quality of life and menorrhagia score. Also, before embolization coagulation analysis will be done and prophylactic antibiotics given. Prophylactic antibiotics will be given via intravenous access 30 min before the UAE procedure. A Foley catheter will be installed for patients before the UAE procedure to keep the bladder empty and prevent obscuration of the uterus by a contrast-filled bladder.

After embolization of uterine arteries, patients will be provided with painkillers, prophylaxis of thromboembolic complications, and encouraged for early activation. Blood sampling for investigation of growth factors will be performed according to the protocol of the study: before the procedure (1), at 1 week (2), 1 month (3), 3 months (4) after the procedure of UAE in the CAD of Women’s Health, UMC. After discharge from the hospital, the members of the study group will observe all patients for the entire study period on an outpatient basis. Ultrasound and/or magnetic resonance imaging will be performed at the Interventional Radiology Department of UMC on the same equipment, by the same physician. MRI will be performed before the procedure in UAE and one year after it, while US scans will be done 1 week after the procedure, and repeated 1 month and 3 months after the UAE procedure on the same US device, by the same physician (Table 1).

#### 2.5.1 Study instruments (questionnaires)

Uterine Fibroid Symptom Health-related Quality of Life Questionnaire (UFS-QOL) and Menstrual Assessment Chart (Menorrhagia score questionnaire) will be utilized for the study.

The UFS-QOL is a tool specifically created to assess the symptoms associated with uterine leiomyoma (26, 27). It was developed by the SIR Foundation and validated in many languages (28). The UFS-QOL tool is currently in use by many researchers . The UFS-QOL contact information and permission to use: Mapi Research Trust, Lyon, France, https://eprovide.mapi-trust.org, No 98290.

The UFS-QOL is a self-reported questionnaire utilized to measure a patient’s objective “pre-treatment and post-treatment symptoms” (e.g., bleeding, cramping) and subjective experience (e.g., a patient feeling “blue” or “less productive”). Information reported in the questionnaire might indicate a patient’s condition without more invasive measures.

Menstrual Assessment Chart (Menorrhagia score questionnaire) is a pictorial method to assess heavy menstrual bleeding (Supplementary material) (29), which was used by many studies assessing blood loss (30).

Both questionnaires will filled by patients before the UAE procedure and repeated 3 and 12 months after the procedure (Table 1).

#### 2.5.2 Blood sampling for growth factors investigation

The blood sampling procedure will be performed for identification of growth factors of interest according to the protocol of the study: before the procedure (1st sample), at 1 week (2nd sample), 1 month (3rd sample), 3 months (4th sample) after the UAE procedure.

#### 2.5.3 Magnetic resonance imaging

The MRI procedures will be performed before the procedure of UAE – initially and one year after UAE (10).

#### 2.5.4 Ultrasound scans

US examination will be done 1 week after the procedure (first control), and repeated 1 month (second control) and 3 months (third control) after the UAE procedure.

### 2.6 Statistical analysis

Descriptive statistics will be used to analyze the study variables (e.g., proportion, mean and standard error, median and interquartile ranges). Statistical methods will include checking assumptions and selecting appropriate tests to compare point estimates and frequencies. Bivariate associations between exposure factors and outcome will be determined by statistical tests (repeated measures ANOVA or Friedman’s test, Pearson’s or Spearman’s correlation coefficients). A linear regression analysis will be used to analyze the associations further to determine which factor best explains the resulting outcome. To adjust for confounders, we will examine associations of all covariates with primary exposure factors and the outcome variable and apply potential confounders to a regression model. Statistical processing of data will be carried out in the software Stata 16.1 (31).

### 2.7 Ethical considerations

The study will follow the standards of the Helsinki Declaration and was approved by the Institutional Ethical Committee of the UMC, protocol#2, date of approval – November 14, 2022.

Participants of the study will be informed about the study’s aims, benefits, possible outcomes, and eventual complications. After the detailed explanation, patients will be asked to sign an informed consent. Support and counseling will be provided throughout the study period. Participation in the study is voluntary, and the study participant will be allowed to leave the study at any stage. If the patient refuses to participate in the study, she will be treated according to the clinical guidelines approved by the Ministry of Health and the UMC clinic. Each patient has the right to refuse to participate in the study without explaining the reasons.

## 3 Anticipated results

### 3.1 Study outcomes

The study hypothesizes that a statistically significant association between blood levels of growth factors (VEGF, TGF-β) in patients before and after UAE will be found.

#### 3.1.1 Primary outcome measures

1. Identify changes in menorrhagia score and Quality of life after uterine artery embolization for uterine fibroids;
2. Define the role of blood levels of growth factors (VEGF, TGF-β) in patients with fibroids before and after uterine artery embolization.

#### 3.1.2 Secondary outcome measures

1. Define the correlation between menorrhagia score and quality of life and blood levels of growth factors (VEGF, TGF-β) before and after the procedure;
2. Outline the correlation between blood levels of growth factors (VEGF, TGF-β) before UAE and fibroid volume;
3. Shape the correlation between fibroid volume after UAE and blood levels of growth factors (VEGF, TGF-β) during follow-up.

### 3.2 Variables to be measured

#### 3.2.1 Study subjects socio-demographic and clinical characteristics

Based on patients’ socio-demographic and clinical data collected during the study subjects’ recruitment, descriptive statistics will be calculated and presented (Table 2). Specific details of Uterine leiomyoma type, nodules count and size (volume), as well as the indication(s) for the UAE procedure, will be analyzed.

**Table 2:** Study subjects’ socio-demographic and clinical characteristics.

Variables	Absolute numbers and percentages
Age	
Ethnicity	
Place of residence	
Education	
Marital status	
BMI	
Menarche	
Gynecological condition(s)	
Gynecological surgeries	
Infection(s)	
Pelvic Inflammatory Disease	
Cervical cytology	
Uterine leiomyoma type	
Uterine leiomyoma nodule(s) size	
Uterine leiomyoma nodules count	
Indications for UAE	
Total	

#### 3.2.2 Analysis of data on patients’ follow-up after the UAE procedure

A specific schedule for study subjects’ follow-up is created to measure anticipated study results (Table 1). UFS-QOL results and menorrhagia scores will be analyzed before, 3 months after, and 1 year after the UAE procedure. Moreover, the structural changes of uterine leiomyoma nodules after UAE will be monitored via a series of US and MRI procedures as presented in Table 3.

**Table 3:** Patients’ follow-up after UAE procedure to register uterine leiomyoma structural changes.

Variables	Follow-up measurements					T-test
	MRI procedure		US procedure			
	Initial	After 1 year	Day 7 after UAE	1 month after UAE	3 months after UAE	
Overall number of myomas						
localization vs. numbers of myomas						
Leading myoma nodule’s localization						
Leading myoma nodule’s size (diameter and volume)						
Leading myoma nodule’s vascularization						
Volume of the uterus						
Uterine artery Doppler flow						

Along with the uterine leiomyoma measurements, blood levels of VEGF and TGF-β will be measured. Possible correlations and associations between (1) changes in UFS-QOL results and menorrhagia scores, (2) structural changes in the uterine leiomyoma nodules, and (3) blood levels of growth (VEGF, TGF-β) factors will be defined and analyzed (Table 4).

**Table 4:** VEGF and TGF-β measurements.

Variables	Follow-up measurements				T-test
	Before UAE	Day 7 after UAE	1 month after UAE	3 months after UAE	
VEGF					
TGF-β					

## 4 Discussion

Considering that uterine leiomyomas present a significant healthcare problem for women and society; the treatment of uterine fibroids is the subject of close attention to both world and national gynecology societies. The pragmatism of radical surgical intervention in situations with an acute clinical picture or gigantic dimensions of fibroids is beyond doubt (32). At the same time, justification for its high frequency in current practice is sometimes questionable. According to experts, even in the US, the number of organ-preserving myomectomies does not exceed 10% among all hysterectomies, and the vaginal approach was identified as the most appropriate whenever feasible (3, 33–36). On the other side, a recently published review evaluated myomectomy done in infertile patients and found that, due to its extremely high adhesiogenic potential, this procedure has been confirmed more harmful than good. Therefore, when myomectomy is performed as an infertility treatment, attention should be drawn to whether the benefits offset the risks (37). Therefore, minimally invasive procedures are of paramount interest.

Furthermore, after the organ-saving operations, the risk of relapse remains high. A recently published retrospective cross-sectional study compared the rates of reappearance and the effect on the quality of life between myomectomy and uterine fibroid embolization in patients diagnosed with uterine leiomyoma (38). The authors reported that there was no notable statistical difference between the two groups, which indicates that both UAE and myomectomy were reasonably successful in preventing uterine fibroid recurrence. These results in line with prior studies that reported similar outcomes for both treatment modalities (39, 40).

Nowadays, it would seem that most practitioners should strive to keep the female reproductive organs. One of the hopes of modern gynecology is the embolization of uterine arteries, which has been widely practiced in modern gynecology since the mid-1990s of the 20th century (41–43). This high-tech surgical procedure is certainly preferable to hysterectomy since it preserves both the organ and its function.

Although UAE was confirmed to be preferable to surgical treatment in patients with symptomatic uterine fibroids (11, 44, 45), it might be accompanied by specific side effects (5) and extremely rare, but life-threatening complications (6).

Embolization of uterine arteries for management of uterine leiomyomas was introduced into Kazakhstani gynecological practice in 2008 at the National Research Center for Maternal and Child Health, Astana. During this period, significant clinical experience was accumulated, and the UAE procedure was proven to be successful. Thus, it can be considered an effective and reliable procedure for leiomyoma treatment (7).

Uterine leiomyoma development is a complex process that involves variety of cytokines and different growth factors; among them, the most important are VEGF and TGF-β (14). Molecular mechanisms of their action are not completely understood and the relationship between their blood levels and fibroid volume is not defined. Currently, many studies on the UAE in women with uterine leiomyomas have been performed worldwide (39, 42, 46). However, blood levels of VEGF and TGF-β in patients before and after UAE are not systematically investigated and the eventual relationship with myoma shrinkage has not been evaluated.

The results of this project will assess menorrhagia score and quality of life improvement after the UAE procedure, and define the association of VEGF and TGF-β blood levels with fibroid shrinkage in patients undergoing UAE, thus, improving knowledge of biochemical markers’ role and approach to clinical management of women with uterine fibroids. The results of this project may become a serious scientific argument for justifying the patient’s choice of UAE as a preferable treatment for uterine fibroids.

### 4.1 Study strengths and limitations

This is the first study comparing blood levels of growth factors (VEGF, TGF-β) with the fibroid volume before and after UAE. Moreover, this study is the first one that links blood levels of VEGF and TGF-β before and after UAE with menorrhagia score and the QoL. This study will assess the value of UAE for myoma shrinkage, menorrhagia score, and QoL of patients undergoing UAE in the local clinical setting. Moreover, this study logistics will allow us to have full insight into patients’ past medical history and clinical data that will facilitate the research outcomes. However, this study has some limitations. Although justified, it has a relatively small sample size. Moreover, the study has a follow-up period of 1 year, which increases the risk of incomplete data collection or missing data in some cases. The third limitation is a relatively short follow-up period of one year, which might limit insight into some parameters/factors that could appear 3 or 5 years after the procedure.

## Ethics statement

The studies involving humans were approved by University Medical Center Institutional Research Ethics Committee. The study will be conducted in accordance with the local legislation and institutional requirements. The participants will provide their written informed consent to participate in this study.

## Author contributions

GA: Conceptualization, Investigation, Methodology, Writing – original draft, Writing – review & editing. GB: Conceptualization, Project administration, Writing – original draft. TU: Conceptualization, Supervision, Writing – original draft. VZ: Conceptualization, Methodology, Writing – original draft. AG: Methodology, Software, Writing – original draft, Writing – review & editing. MT: Conceptualization, Methodology, Project administration, Resources, Writing – original draft, Writing – review & editing.
