# Supplementary material for: The role of VEGF and TGF-β blood levels for fibroid shrinkage, menorrhagia score, and quality of life improvement after uterine artery embolization for uterine fibroids: a study protocol
Source: Front Med (Lausanne). 2024 Aug 6;11:1382822. doi: 10.3389/fmed.2024.1382822 (PMC11334076; doi:10.3389/fmed.2024.1382822)
Supplement: Supplementary file 1 [file Image_1.pdf]

Menstrual Assessment Chart

Date of start __________________

Day	1	2	3	4	5	6	7	8	9	10	11	12
Pads												
Lightly soaked 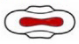												
Moderately soaked 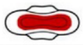												
Heavily soaked 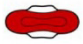												
Clots (small or large)												
Tampons												
Lightly soaked 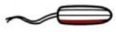												
Moderately soaked 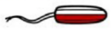												
Heavily soaked 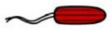												
Clots (small or large)												
Score												

Total score: _________

Directions
Keep a tally of the number of pads or tampons you use each day of your cycle and their level of saturation. Also take note of clots or overflow. Clots >1 cm in size are considered large.

Scoring
Pads (score per pad)
Lightly soaked: 1 point
Moderately soaked: 5 points
Heavily soaked: 20 points

Tampons (score per tampon)
Lightly soaked: 1 point
Moderately soaked: 5 points
Heavily soaked: 10 points

Clots
Small: 1 point
Large: 5 points

INTERPRETATION
A score of ≥ 100 points indicates probable menorrhagia.

Source: Jenny M. HIGHAM, P. M. S. O'BRIEN, R.W. SHAW, Assessment of menstrual blood loss using a pictorial chart, British Journal of Obstetrics and Gynaecology, August 1990, Vol. 97, pp. 734-739
